# The Role of Superabsorbent Polymer on Strength and Microstructure Development in Cemented Dredged Clay with High Water Content

**DOI:** 10.3390/polym10101069

**Published:** 2018-09-27

**Authors:** Xia Bian, Lingling Zeng, Yongfeng Deng, Xiaozhao Li

**Affiliations:** 1 Key Laboratory of Ministry of Education for Geomechanics and Embankment Engineering, Hohai University, Nanjing 210098, China; bianxia2010@gmail.com; 2 Institute for Underground Space and Geoenvironment, School of Earth Sciences and Engineering, Nanjing University, Nanjing 210046, China; lixz@nju.edu.cn; 3 Institute of Geotechnical Engineering, College of Civil Engineering, Fuzhou University, Fuzhou 350116, China; 4 Institute of Geotechnical Engineering, School of Transportation, Southeast University, Nanjing 210096, China; noden@seu.edu.cn; 5 Sino Probe Center, Chinese Academy of Geological Sciences, No. 26 Baiwanzhuang Street, Beijing 100037, China

**Keywords:** superabsorbent polymer, cemented clay, microstructure, SEM, high water content

## Abstract

This paper presents the role of superabsorbent polymer (SAP) on strength and microstructure development in cemented clays with notably high water content. A series of unconfined compressive strength (UCS), scanning electron microscope (SEM) and X-ray diffraction (XRD) tests were performed to identify strength behavior and microstructure. Results showed that SAP significantly influenced the mechanical behavior of cemented clays with notably high water content, characterized by an increase in the unconfined compressive strength and a decrease in the after-curing water content with SAP content. This revealed that the strength increase due to SAP was directly related to the water absorption by SAP. Meanwhile, XRD results showed that the hydration products were controlled by cement and lime content, regardless of SAP content. That meant there was no chemical reaction between SAP particles used in this study and cement or lime. The microstructure analysis by SEM revealed that SAP played an important role in the microstructure of cemented clays. With an increase in SAP content, the water absorbed by SAP increased significantly, leading to a decrease in the pore volume and a denser soil fabric. This behavior indicated that the primary role of SAP on strength increase was to absorb and fix water in cemented clays. Consequently, the clay–cement cluster distance decreased with an increase in solid mass (soil particles and swollen SAP particles) and a decrease in pore water. The corresponding tighter flocculated fabric due to SAP eventually led to the strength increase.

## 1. Introduction

During the process of dredging from rivers, ports, and harbors, thousands of tons of dredged clays are generated each year. For example, by 2015, more than 14 billion m^3^ of clay materials had been dredged annually from main rivers and commercial ports in China [1]. The number of dredged clays from waterways in the United States was approximately 300 million m^3^ per year [2,3]. The dredged clays were often classified as waste materials, dumped in the ocean or in a storage yard [4,5]. Therefore, the large amount of dredged clays may cause severe environmental problems. On the other hand, many natural materials (i.e., rock, sand, and soil) were used as raw material for infrastructure construction, which also increased the strain on the natural environment. From an environmentally friendly perspective, making these waste dredged clays useful as construction raw materials would not only significantly ease the strain on the environment, but also provide a more sustainable supply of the raw materials used in construction.

It is noteworthy that several studies focused on the re-use of clays as cement substitute [6] or aggregates [7]. Another common method observed was to use stabilizing binders such as cement or lime for improving the mechanical behavior of dredged clays to transform these wastes into construction materials. The chemical reaction between cement/lime, water, and clays resulted in a hydration of the cement, followed by the pozzolanic reaction between hydrates and silica in the clays. These cementitious products formed during the physico-chemical reaction process and provided the artificial cementation within the clay particles, which enhanced the strength and reduced the compressibility of the dredged clays [8]. In general, cemented dredged clays can be considered to comply with construction standards and long-term environmental requirements.

The microstructure formed during the physico-chemical reaction played a decisive role in the strength of the cemented dredged clays. As observed by several researchers [9,10,11,12,13], the increase of water content, or the decrease of cement content, led to the increase of porosity and macropores, and eventually the relatively looser bond fabrics with clay particles, resulting in the decrease in strength. The typical water content of dredged clay in storage yards in China was approximately two or three times the liquid limit [1,14,15,16]. Hence, the effectiveness of the cement mixing method for these dredged clays with notably high water content was expected to be limited. In other words, large amounts of cement material or additional additives were expected to be consumed to improve the mechanical behavior of the dredged clays with notably high water content in order to meet the construction standards.

To improve the cementation effectiveness, many binder materials, such as fly ash, slag, and organic polymer, have been introduced into the cement mixing method to improve the mechanical behavior of cemented dredged clays [17,18,19,20,21,22,23]. Specifically, organic polymers have been used as a soil stabilizer to improve the strength characteristics of sandy materials, which refined the loose structure and improved the strength characteristic of raw materials [21,22,23]. However, it should be emphasized that these common additional binders had little effect on consuming water, which appeared to be the primary factor that affected the strength increase in cemented dredged clays with notably high water content. Specifically, the use of organic polymers was mainly focused on the dry materials such as sand. Therefore, to deal with these particular problematic clays with high water content, a water-absorbent agent such as superabsorbent polymer (SAP) is of great interest. A common water absorbent with excellent water absorption, SAP has been used as a water-saving agent in the agricultural industry [24] or as a water reservoir to improve the self-desiccation of concrete [25,26,27,28,29]. For the purpose of increasing the strength characteristic of cemented dredged clay with notably high water content and lowering the cost, Bian et al. [30,31,32] introduced SAP into the cement mixing method as a water-reducing agent to improve the strength properties of cemented clay with notably high water content. Based on a series of experimental studies, Bian et al. [30,31,32] showed that SAP had a significant positive effect on the mechanical behavior improvement of cemented clay with notably high water content. The unconfined compressive strength increased with the increase in SAP content, whereas the compressibility decreased with the increase in SAP content.

It has been recognized that for cemented clay or concrete, the changes in engineering properties were directly related to the change in microstructure [12,29]. However, the role of SAP on microstructure development, which might be of great importance to understand the mechanism of strength development, is not available for cemented dredged clays.

This study aims to investigate the role of SAP on microstructure changes in cemented dredged clays with notably high water content to explain the different degrees of strength development according to influential factors such as SAP content, lime content, water content, and curing time. First, the influence of SAP on the unconfined compressive strength was experimentally investigated. Then, the corresponding microstructure was identified using a scanning electron microscope and through X-ray diffraction tests. Finally, the variations in the microscopic results of mineral composition, pore structure and morphology with SAP were used to understand the mechanism of SAP on the strength increase of cemented dredged clays with notably high water content.

## 2. Materials and Methods

### 2.1. Materials

The soil used in this study was Kemen clay, reported by Hong et al. (2013), which was the dredged clays with high water content obtained from the harbor construction at the city of Fuzhou, Fujian Province. The liquid and plastic limit of Kemen clay is 60% and 30%, respectively, and it can be classified as CH clay, according to the Unified Soil Classification System [33]. Based on the XRD analysis, Kemen clay consisted of 54% illite, 16% kaolinite, 15% chlorite, and 15% smectite. The basic physical properties of Kemen clay are listed in Table 1 [34].

The ordinary type of Portland cement and quick lime were used as stabilizing binders in this study. The main properties of this cement and lime are presented in Table 2.

**Figure 1:**
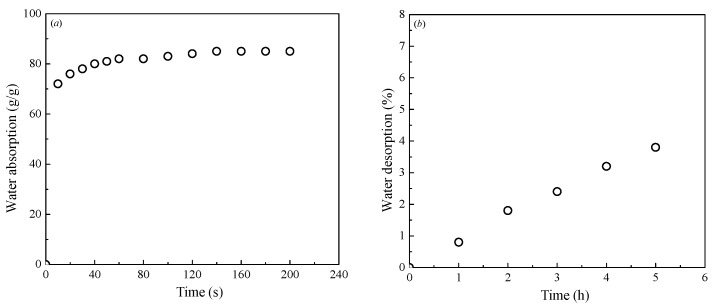
Time dependent water absorption and desorption behavior of the superabsorbent polymer (SAP) used: (**a**) water absorption, (**b**) water desorption.

The properties of the superabsorbent polymer (SAP) used in this study are listed in Table 3. The SAP used was made of a suspension-polymerized, covalently cross-linked acrylamide/acrylic acid copolymer with a dry-bulk density of 800 kg/m^3^. Under ambient laboratory conditions, the SAP particle consisted of white powder without water and transmitted into gel state after absorbing water. The water retention ability of SAP was measured by the tea bag method, as shown in Figure 1. It is clear that the SAP used in this study possessed high water absorption capacity with a good water retention property and a low desorption rate.

### 2.2. Sample Preparation

The Kemen clay were first mixed with a predetermined quantity of water to achieve a clay slurry with the water content at approximately two or three times the liquid limit (120%, 150%, and 180%), to simulate the typical state of dredged clay from disposal ponds in China. Afterwards, SAP particles were poured and mixed with clay slurry for water absorption. Then, the pre-absorbing SAP–clay slurry was mixed with cement and lime powder for approximately 10 min to achieve uniformity. In this study, the cement content *A*_C_ was 3% by weight of dry soil. The lime content *A*_L_ was 7% and 12% by weight of dry soil. To investigate the effect of SAP, different amounts (1‰, 5‰, and 10‰) of SAP content *A*_p_ were used. The cemented clay paste was cured in containers with a diameter of 50 mm and a height of 100 mm. After 24 h, the cylindrical samples were dismantled. All cylindrical samples were then wrapped in open plastic bags and cured in a controlled environment (20 ± 2 °C and 95% relative humidity) until the different curing times lapsed (7, 28, and 90 days). The detailed experimental program is shown in Table 4.

### 2.3. Testing methods

#### 2.3.1. Unconfined Compressive Strength

Unconfined compressive strength was determined through unconfined compression tests (UCT) performed on samples after 7, 28, and 90 days of curing. The UCT was performed based on the ASTM D4219 standard [35] using the cylindrical samples with a diameter of 50 mm and a height of 100 mm. Triplicate measurements were conducted for the after-curing water content and unconfined compressive strength (*q*_u_), and the average values were reported. The rate of vertical displacement in the unconfined compression tests was 1 mm/min.

#### 2.3.2. After-Curing Water Content

The after-curing water content (*w*_t_) represents the water content of cemented clays at certain curing times, which has been recognized as an important parameter for analyzing the mechanical behavior of cemented clays [36]. In this study, the amount of water absorbed by SAP transmitted into SAP gel and filled up the large pores [30]. Hence, the pore volume of cemented clays does not depend on the swollen SAP. In other words, the after-curing water content should be determined as the water content between clay particles, contributing to the pore volume. Therefore, before water content determination, the swollen SAP particles were carefully removed. Then, the after-curing water content of sample without swollen SAP was determined by oven drying the cemented clay specimens to 105 °C for at least 24 h.

#### 2.3.3. Microstructure

The X-ray diffraction (XRD) test and scanning electron microscope (SEM) test were carried out to investigate the microstructural characteristics of cemented dredged clays with SAP. The lyophilized specimen was prepared for the SEM test. First, the cemented clays were cut into small cubes (1 cm × 1 cm × 1 cm). Then, the small specimens were submerged into liquid nitrogen and vacuum cooled to approximately −210 °C. Finally, the frozen samples were put into the vacuumed chamber of a freeze dryer for sublimation for approximately 24 h. It has been proven that this procedure minimizes the disturbance of the soil microstructure [37,38,39].

To perform the SEM analysis, the lyophilized specimen prepared according to the above-mentioned method was coated with a gold layer to induce conductivity. Then, the SEM analysis of these samples was conducted using a LEO1530VP (One Zeiss Drive, Thornwood, NY, US) scanning electron microscope.

The X-ray diffraction analysis was conducted by using a Rigaku D/Max-2500 (Rigaku Beijing Corp, Hong Kong, China.) X-ray diffractometer equipped with a copper anticathode. The lyophilized samples were first milled into fine powders, and were scanned for a two-theta (2θ) value ranging between 5° to 60° with a step length of 0.02° and a scanning rate of 2°/min. The results were semi-qualitatively analyzed using JADE 5.0. Material Date, Inc (Livermore, CA, US).

## 3. Results and Discussions

### 3.1. Unconfined Compressive Strength

**Figure 2:**
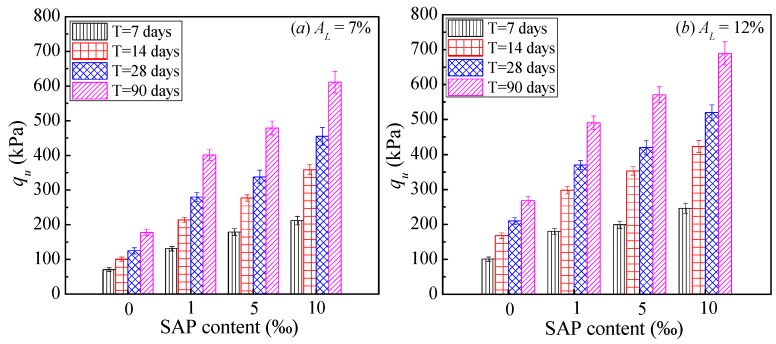
Effect of SAP content on *q*_u_ (w_0_ = 120%).

**Figure 3:**
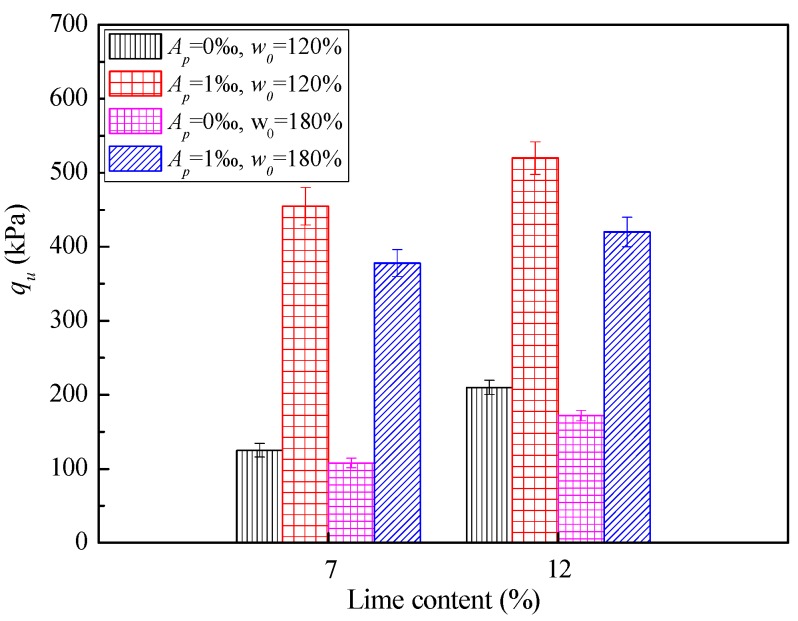
Effect of lime content on *q*_u_ (curing of 28 days).

**Figure 4:**
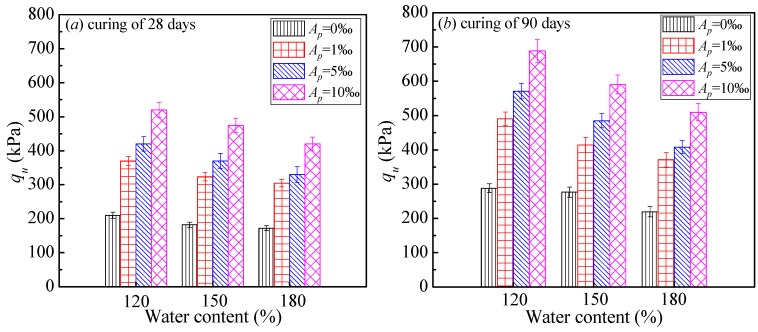
Effect of initial water content on *q*_u_ (*A*_L_ = 12%).

Figure 2 depicts the typical relationship between SAP content and the unconfined compressive strength (*q*_u_) of cemented dredged clays at an initial water content of *w*_0_ = 120%. With the increase in SAP content, the unconfined compressive strength (*q*_u_) increased significantly at a given lime content and curing time. Specifically, when 1‰ of SAP content was added to cemented dredged clays with notably high water content, *q*_u_ increased by an average of 0.8 times compared to those without SAP. For the case of 5‰ and 10‰ SAP, the increase in *q*_u_ reached approximately 1.3 and 1.9 times compared to those without SAP, respectively. The significant increase in unconfined compressive strength emphasized that SAP had a positive influence on the strength of cemented dredged clays at notably high water content, with significant improvement on the effectiveness of the cemented mixing method for this specific case. Figure 3 and Figure 4 show the effect of lime content and initial water content on unconfined compressive strength (*q*_u_), respectively. As expected, for a given SAP content and initial water content, *q*_u_ increased with the increase in lime content, due to the increase in cementitious products. Inversely, for a given SAP content and lime content, *q*_u_ decreased with the increase in lime content, due to the looser microstructure with higher water content. These observations agree with the results of previous experimental findings [9,12,13].

**Figure 5:**
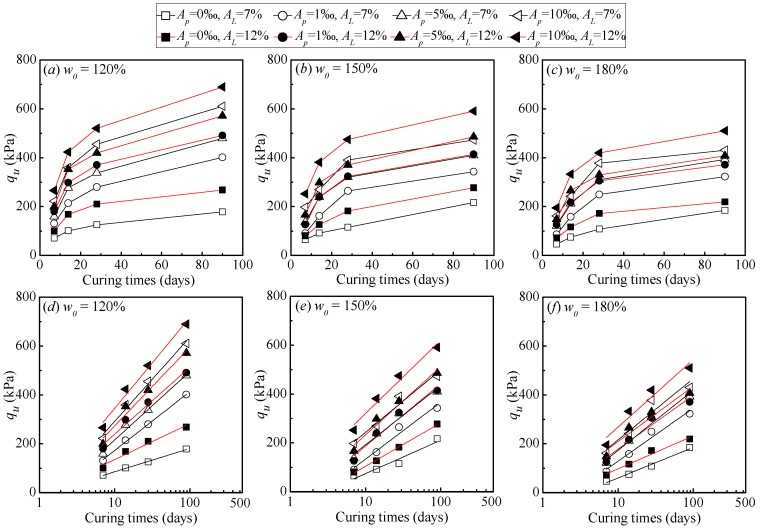
Strength development with curing times.

The strength development with curing time for cemented dredged clays with SAP is plotted in Figure 5. It is evident that for a given lime content and initial water content, the strength development curve of a specimen with higher SAP content is found above that with lower ones. This indicated that the positive effect of SAP on strength increase was expected to exist throughout all the curing periods (from 7 to 90 days). At the early stage (7, 14 days), the strength increase of cemented clays was due to the hydration process of cement, with the significant increase in cementitious products and the consummation of water. It should be emphasized that with the presence of SAP, a higher amount of pore water in the system of dredged clays was fixed and transformed into a swollen SAP particle approximately three times in size [30] due to the greater absorption ability of SAP as shown in Figure 1. As a result, the soil structure of cemented clays with high water content were expected to be significantly altered, with a significant decrease in pore water or pore volume with the increase in SAP content, leading to a denser microstructure of cemented clays. As the curing period increased, the role of SAP in strength increase continued, indicating that the desorption of SAP was limited as shown in Figure 1, with almost no negative influence on the microstructure of cemented clays with high water content in this study. In other words, the absorption potential of SAP played a decisive role in the strength increase for cemented clays with notably high water content, whereas the low desorption rate had almost no influence on the strength increase at the longer times. In addition, the strength development with curing times in the natural logarithmic scale can be expressed as a linear variation. The slope of these linear relations represented the rate of strength development with time. It can be observed that for a given lime content and water content, the rate of strength development with time showed a slight increase tendency with SAP content. This behavior also emphasized the efficiency of the strength increase due to the presence of SAP.

**Figure 6:**
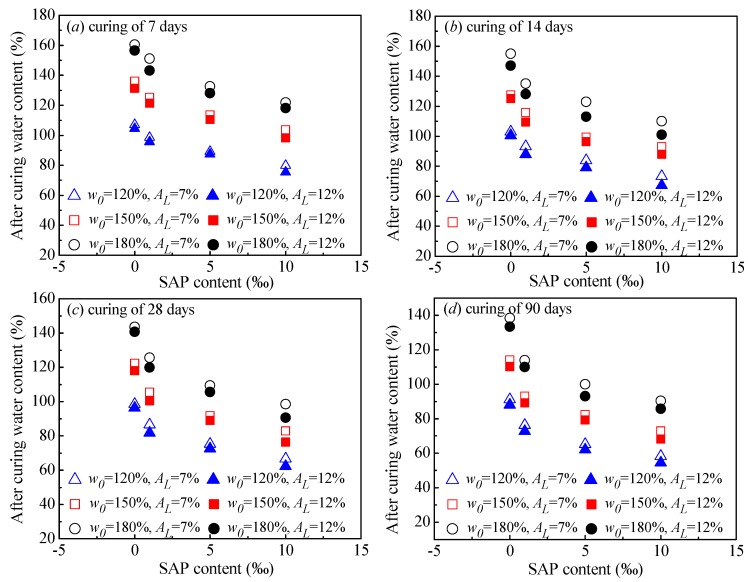
Variation of after curing water content with SAP content.

To further analyze the role of SAP in strength increase, the after-curing water content (*w*_t_) was used as the indicator of the water absorption of SAP. Figure 6 shows the effect of the SAP content on *w*_t_. Note that the water absorbed by SAP transformed to swollen SAP particles, which were thought to be solid parts, filling up the large voids inside the cemented clays. Hence, the after-curing water content (*w*_t_) defined in this study was the water content of the specimen without SAP particles. Therefore, the swollen SAP particles were removed carefully before the water content determination. Although the SAP particle removal process could result in a certain degree of error (a certain proportion of swollen SAP particles remained inside the specimen during water content determination), the relationship of the after-curing water content (*w*_t_) with SAP content (*A*_p_) still showed a clear decrease tendency. This behavior suggests that the role of SAP in cemented clay with high water content is to absorb water content in cemented clay and to fill up the large voids, leading to a denser microstructure [30].

**Figure 7:**
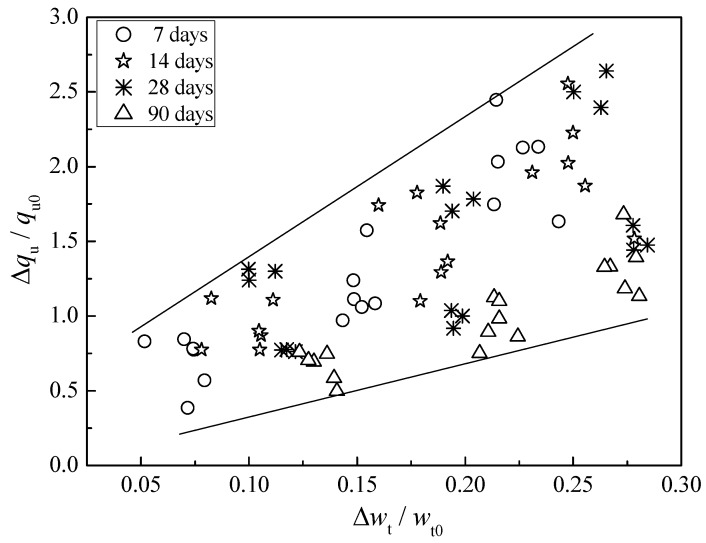
Effect of water absorption on strength increase.

Figure 7 shows the effect of water absorption on strength increase. Note that *w*_t_ and *w*_t0_ represent the after-curing water content of cemented clays with SAP and without SAP, respectively. Hence, the water absorption due to SAP can be defined as Δ*w*_t_ = *w*_t_ − *w*_t0_. Meanwhile, *q*_u_ and *q*_u0_ are defined as the unconfined compressive strength of cemented clays with SAP and without SAP, respectively. The corresponding strength increase can be expressed as Δ*q*_u_ = *q*_u_ − *q*_u0_. As shown in Figure 7, there was a clear tendency of strength increase with the water absorption due to SAP. This indicated that for the dredged clays with notably high water content, the strength increase of cemented clays with SAP was mainly due to the water absorption of SAP. In other words, with the increase of SAP content, a higher amount of water fixed by SAP particles, which in turn transformed into the swollen SAP particles, occupied the pore spaces or reduced the pore volume, consequently resulting in the strength increase. In the sections below, the mechanism of strength development due to SAP will be correlated with the microstructure investigation.

### 3.2. Microstructure Analysis

#### 3.2.1. Results of XRD Tests

**Figure 8:**
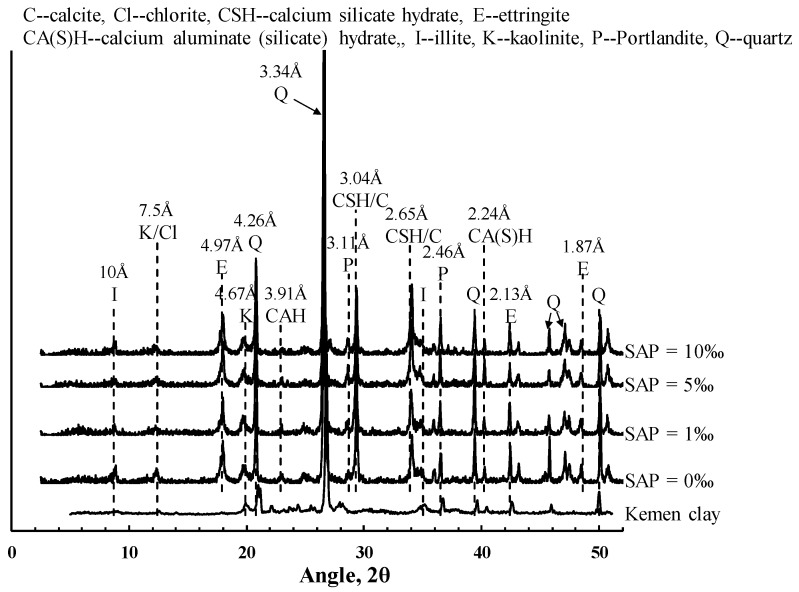
XRD diffractograms of cemented clays with different SAP content.

The X-ray diffraction patterns of the untreated Kemen clay and cemented clay with various SAP content at 28 days are presented in Figure 8. Results show that the main minerals for untreated Kemen clay are quartz and clay minerals, such as calcite, kaolinite, illite, and chlorite, which is consistent with that reported in Hong et al. (2013).

When cement and lime were added to the Kemen clays, some new reflection corresponding to the composition of hydration products were detected as Portlandite, CSH, calcium aluminate (silicate) hydrate (CA(S)H), and ettringite. These hydrate products have also been identified in cement- and lime-treated clays by several researchers [18,19,20].

The new reflection corresponds to calcium silicate hydrate formed Ca_1.5_SiO_3.5_•xH_2_O (PDF file 033-0306) or 5CaO•3SiO_2_•2H_2_O (PDF file 012-0475) that appeared at d-space at 3.04 and 2.65 Å at 2θ = 29.4° and 33.9°. The appearances of calcium aluminate hydrate formed Ca_3_Al_2_O_6_•xH_2_O (PDF file 002-0083) and calcium aluminate silicate hydrate formed Ca_3_Al_2_SiO_4_(OH)_8_ (PDF file 032-0151) are located at d-space at 3.91 and 2.24 Å at 2θ = 22.8° and 40.2°, respectively. Moreover, the appearance of ettringite formed Ca_6_Al_2_(SO_4_)_3_(OH)_12_•26H_2_O (PDF file 041-1451) can be identified at d-space at 4.97, 2.13, and 1.84 Å at 2θ = 17.9°, 42.4°, and 48.6°.

By comparing the XRD results at different SAP contents, it can be observed that the intensity of the hydration products (i.e., CSH, CA(S)H, and ettringite) remained almost unchanged. This indicates that the SAP used in this study did not participate in the chemical reactions between the cement, lime and soil particles. Hence, the hydration products did not vary with SAP content for a given cement and lime content. This behavior implies that the primary role of SAP in cemented clays with notably high water content was to refine the soil matrix fabric, rather than to enhance the cementation bonding. In the sections below, SEM images will be used to confirm this deduction.

#### 3.2.2. Effect of SAP

**Figure 9:**
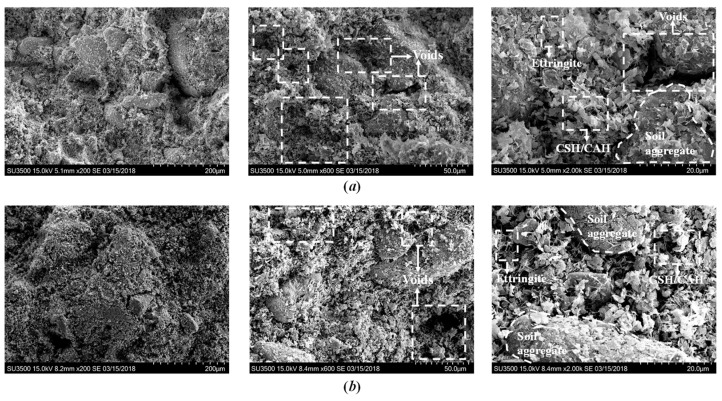
Scanning electron microscope (SEM) images of cemented dredged clays with (**a**) 0‰ SAP and (**b**) 1‰ SAP after 7 days of curing.

Figure 9 shows the microstructure of cemented clays with 0‰ and 1‰ SAP after 7 days of curing (*w*_0_ = 120%, *A*_L_ = 12%). As reported in previous studies [40,41], the reticulate and foil-like CSH and needle-like ettringite could be clearly identified from the cemented clays, which represented the typical cementitious products formed during the physico-chemical reaction process between the cement, lime, and soil particles. Moreover, it was observed that the reticulate and needle-like hydration products covered the surface of soil aggregates and occupied the large pores between the soil aggregates, which formed a well-developed flocculated fabric inside the cemented clays with notably high water content. The formation of flocculated fabric eventually led to the enhancement of the soil aggregate cementation bond and resulted in the strength increase for the cement mixing method [41,42].

**Figure 10:**
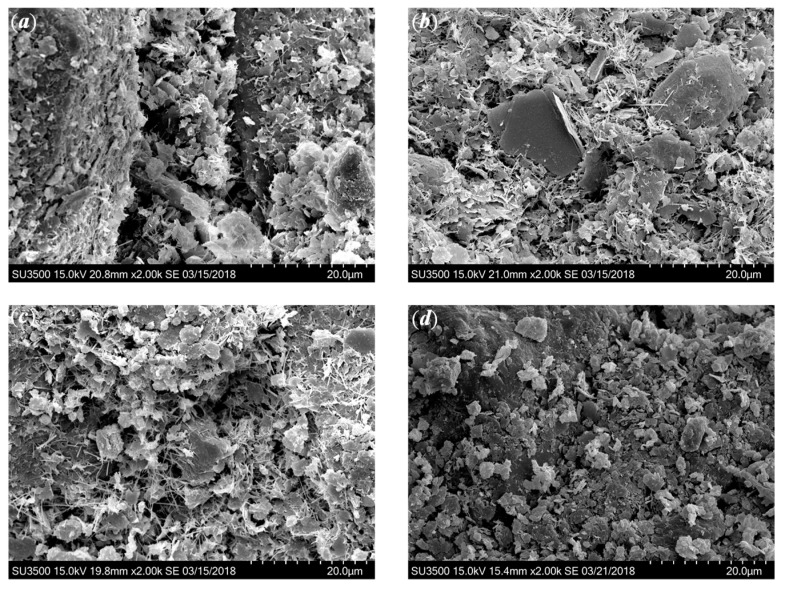
SEM images showing the effect of SAP: (**a**) *A*_p_ = 0‰, *A*_L_ = 12%, 28 days; (**b**) *A*_p_ = 1‰, *A*_L_ = 12%, 28 days; (**c**) *A*_p_ = 0‰, *A*_L_ = 12%, 90 days; (**d**) *A*_p_ = 1‰, *A*_L_ = 12%, 90 days.

Regarding the effect of SAP on the microstructure of cemented clays, when no SAP added as shown in Figure 9a, more pore space was found between the soil aggregates and the stacked hydration products, due to the fact that the initial water content was too high to be consumed by the amount of cement and lime used in this study. Hence, the formation of the soil fabric without SAP was relatively looser. For the sample with 1‰ SAP as shown in Figure 9b, the pore volume was reduced significantly as compared with the case without SAP. Therefore, the denser state of microstructure was formed when SAP was added to the cemented clays with notably high water content as a water absorption agent. A comparison between Figure 9a,b illustrates that the role of SAP was to absorb and fix the water that remained inside the soil matrix, and was transformed into solid swollen SAP particles. This process reduced the pore volume inside the soil matrix, resulting in a denser microstructure, eventually leading to the increase in strength (see Figure 2). In addition, the microstructure of cemented clays at different curing times as shown in Figure 10 confirmed that the denser microstructures were observed in the cemented clays with SAP at longer curing times. Therefore, for the same amount of cementitious products (the same cement and lime content as shown in Figure 8), the soil fabric formation played a significant role in the strength increase, that is, the higher strength was attributable to the lower pore volume and the denser microstructure.

#### 3.2.3. Effect of Lime Content

**Figure 11:**
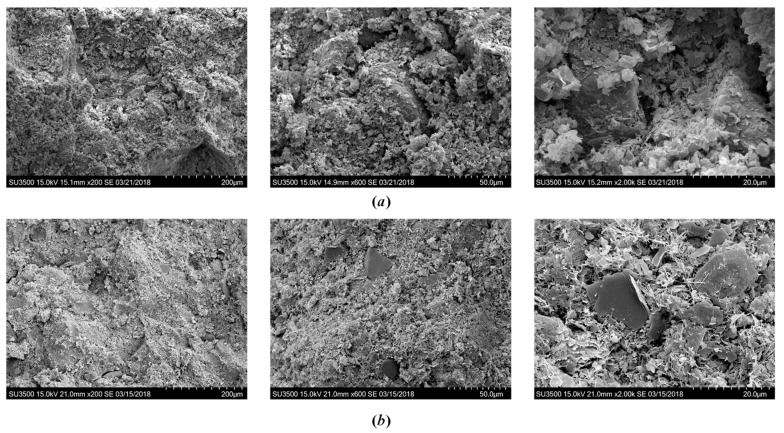
SEM images showing the effect of lime content after 28 days of curing: (**a**) *A*_p_ = 1‰, *A*_L_ = 7% (**b**) *A*_p_ = 1‰, *A*_L_ = 12%.

Figure 11 depicts the SEM images of cemented clays with SAP with different lime content. As the lime content increased, the hydration products significantly increased. Meanwhile, these additional cementitious products also filled up the pores, which resulted in a relatively denser soil fabric for the sample with higher lime content. This behavior emphasized that the strength increase from higher lime content was due to both the enhancement of cementation bonding (more hydration products) and the decrease of pore volume (denser fabric).

#### 3.2.4. Effect of Initial Water Content

**Figure 12:**
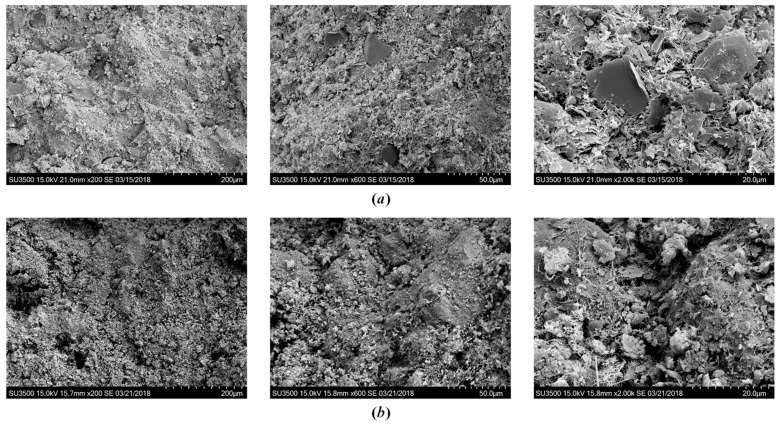
SEM images showing the effect of initial water content after 28 days of curing: (**a**) *w*_0_ = 120%, (**b**) *w*_0_ = 180%.

Figure 12 shows SEM images of cemented clays with 1‰ SAP and 12% lime content with different initial water content after 28 days of curing. It is evident that the intensity of hydration products for the sample with the higher initial water content was significantly less than that with the lower initial water content. In addition, the initial water content also influenced the pore volume and the higher water content resulted in larger pore volume, leading to the looser state of microstructure. The microstructure change with the initial water content was similar to the effect of SAP. SAP particles absorbed the water inside the clay slurry, which was equivalent to reducing the initial water content, resulting in a denser state of microstructure. Hence, for a given cement and lime content, SAP content increased the strength improvement, and thus enhanced the effectiveness of the cement mixing method.

#### 3.2.5. Effect of Curing Times

**Figure 13:**
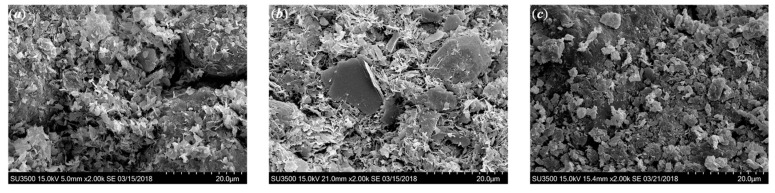
SEM images of cemented dredged clays with 1‰ SAP at different curing times: (**a**) 7 days, (**b**) 28 days, and (**c**) 90 days.

The effect of curing time on the microstructure is illustrated in Figure 13. It was found that the hydration products tended to increase over time due to continuous cementation reactions. With the increase in hydration products, the cementation bonding between soil aggregates and cement clusters tended to be stronger. The large pores were also filled by the increasing hydration products, which led to the decrease in pore volume and a denser state. This behavior is similar to the curing time effect on strength increase for cemented clays [12].

### 3.3. Mechanism of SAP on Strength Development

This investigation confirms that the strength increase of cemented clays due to SAP is directly related to the microstructure changes. As shown in Figure 8, the intensity of cementitious products with a different SAP content, which formulates the new structure/bonding, is almost identical for the cemented clays at a given cement and lime content [12,36]. Hence, the mechanism of SAP on strength increase can be illustrated as follows: when SAP particles were added to the clay slurry, the volume of swollen SAP particles noticeably increased and, accordingly, the solid mass (soil particles and swollen SAP particles) increased with a decrease in pore water. In other words, the presence of SAP resulted in a significant decrease in pore volume with the increased size of the swollen SAP particles due the greater absorption potential as shown in Figure 1. After cement and lime were mixed into the clay–SAP slurry, the hydration products enveloped the soil aggregates and the swollen SAP particles and filled up the large pores. Hence, the distance between clay–cement clusters significantly decreased, resulting in a denser flocculated fabric within the soil matrix (as shown in Figure 9 and Figure 10). When there was no SAP, the clay–cement clusters tended to form large pores, leading to a looser and open structure. At the longer times, there could be some water detached with SAP due to the desorption nature of SAP. However, the stronger cementation bonding between soil aggregates and cement clusters tended to be the main contributions to the strength increase over the long term. Meanwhile, the low desorption rate may not influence the soil fabric with stronger cementation bonding as shown in Figure 12. Therefore, a higher strength increase in cemented clays still can be observed for those with SAP compared to those without SAP. In summary, the strength increase due to SAP for cemented clays with notably high water content was primarily attributed to the improvement of the soil fabric due to the significant decrease in water absorption by SAP and the low desorption rate at long curing times.

## 4. Conclusions

This paper presents the role of SAP on the strength development of cemented clays with notably high water content by incorporating the changes in soil microstructure identified using scanning electron microscope and XRD analysis. The following conclusions can be drawn from this study:

The unconfined compressive strength of cemented clays significantly increases with SAP content. Inversely, the after-curing water content significantly decreases with SAP content. Further examination shows that the strength increase due to SAP is attributed to the amount of water absorbed by SAP for the cemented clays with notably high water content.

XRD results indicate that the main hydration products for the cemented clays with SAP are CSH, CA(S)H, and ettringite. The intensity of hydration products shows no variation with SAP content, indicating that SAP has not participated in the chemical reactions between cement, lime, and soil particles.

SEM results reveal that with an increase in SAP content and a decrease in initial water content, the pore volume decreases and the soil fabric becomes denser. Meanwhile, with the increase in lime content, the hydration products increase with a decrease in pore volume. The effect of curing time is mainly attributable to the cementation bonding developed during cementation reactions and the further reduction in pore volume.

The primary mechanism of strength increase caused by SAP in cemented clays with notably higher water content is that when SAP is added, the solid mass (soil particles and swollen SAP particles) increases with a decrease in pore water, resulting in a lower pore volume and a denser microstructure due to the water absorption. This alteration in microstructure due to SAP eventually leads to the strength increase.

## Figures and Tables

**Table 1:** Basic physical properties and mineralogical composition of Kemen clay.

Soils	G_s_: Mg/m^3^	Liquid Limit: %	Plastic Limit: %	Activity	Illite	Chlorite	Kaolinite	Smectite
					Composition (%)			
Kemen Clay	2.67	60.0	30.0	1.20	54	15	16	15

**Table 2:** Composition of Portland cement and lime used in this study (%).

Binders	CaO	SiO_2_	Al_2_O_3_	Fe_2_O_3_	SO_3_	MgO	CO_2_	Other
Cement	59.3	22.41	4.24	3.46	4.11	3.48	-	3.0
Lime	95.3	0.06	-	-	-	2.13	0.33	2.18

**Table 3:** Properties of the superabsorbent polymer.

Particle Size (μm)	Water Absorption (g/g)	Water Desorption (%)
120–150	85	3.8

**Table 4:** Test program.

Initial Water Content (%)	Cement and Lime Content, *A*_A_ = *A*_C_ + *A*_L_ (%)	SAP Content, *A*_p_ (‰)	UCT ^a^	XRD	SEM
120	15	0	7 D, 14 D, 28 D, 90 D ^b^	28 D	7 D, 28 D, 90 D
120	15	1	7 D, 14 D, 28 D, 90 D	-	-
120	15	5	7 D, 14 D, 28 D, 90 D	-	-
120	15	10	7 D, 14 D, 28 D, 90 D	28 D	7 D, 28 D, 90 D
120	10	0	7 D, 14 D, 28 D, 90 D	-	-
120	10	1	7 D, 14 D, 28 D, 90 D	-	-
120	10	5	7 D, 14 D, 28 D, 90 D	-	-
120	10	10	7 D, 14 D, 28 D, 90 D	28 D	28 D
150	15	0	7 D, 14 D, 28 D, 90 D	-	-
150	15	1	7 D, 14 D, 28 D, 90 D	-	-
150	15	5	7 D, 14 D, 28 D, 90 D	-	-
150	15	10	7 D, 14 D, 28 D, 90 D	-	-
150	10	0	7 D, 14 D, 28 D, 90 D	-	-
150	10	1	7 D, 14 D, 28 D, 90 D	-	-
150	10	5	7 D, 14 D, 28 D, 90 D	-	-
150	10	10	7 D, 14 D, 28 D, 90 D	-	-
180	15	0	7 D, 14 D, 28 D, 90 D	-	-
180	15	1	7 D, 14 D, 28 D, 90 D	-	-
180	15	5	7 D, 14 D, 28 D, 90 D	-	-
180	15	10	7 D, 14 D, 28 D, 90 D	28 D	28 D
180	10	0	7 D, 14 D, 28 D, 90 D	-	-
180	10	1	7 D, 14 D, 28 D, 90 D	-	-
180	10	5	7 D, 14 D, 28 D, 90 D	-	-
180	10	10	7 D, 14 D, 28 D, 90 D	-	-

Note: ^a^ partial unconfined compression tests (UCT) results have been reported in Bian et al. (2016, 2017a, b); ^b^ 7 D, 14 D, 28 D, 90 D indicates that the corresponding tests were conducted for the sample curing after 7, 14, 28, and 90 days, respectively.
